# International Survey Regarding the Use of Rehabilitation Modalities in Horses

**DOI:** 10.3389/fvets.2018.00120

**Published:** 2018-06-11

**Authors:** Janine M. Wilson, Erica McKenzie, Katja Duesterdieck-Zellmer

**Affiliations:** ^1^Oregon Equine, Damascus, OR, United States; ^2^Department of Clinical Sciences, Carlson College of Veterinary Medicine, Oregon State University, Corvallis, OR, United States

**Keywords:** sports medicine, tendon, ligament, performance, exercise, rehabilitation, survey

## Abstract

To define which biologic, electrophysical and other modalities are used in horses for injury or performance issues, a questionnaire regarding 38 modalities was distributed to eight veterinary groups. A total of 305 complete or partial responses were obtained from over 10 geographic regions; 75.4% from private equine practice or regional private equine referral hospitals, 14.1% from university teaching hospitals or satellite clinics, 8.2% from private mixed animal practice, and 2.3% from veterinary rehabilitation centers. The majority of respondents were located in the USA (60%), Europe (25.6%), and Canada (5.6%). Respondents reported working with athletic horses primarily in the disciplines of hunter-jumper (26.9%), dressage (16.0%), and pleasure riding (14.7%), followed by Western riding, track racing, and eventing. Warmbloods (39.7%) were the predominant breed presenting to respondents, followed by Thoroughbreds (20.3%) and Quarter Horses (17.3%) ahead of other breeds. All 38 modalities were used by respondents. The 10 most prominently utilized were controlled hand walking (97.3%), therapeutic shoeing (96.1%), ice (95.2%), compression bandaging (89.5%), platelet rich plasma (PRP; 86.5%), therapeutic exercises (84.3%), interleukin-1 receptor antagonist protein therapy (IRAP; 81.4%), stretching (83.3%), and cold water hydrotherapy (82.9%). Heat (77.6%), massage (69.0%), and acupuncture (68.3%) were also commonly utilized. The least prominently used modalities were hyperbaric oxygen therapy (9.4%), cytowave (8.3%), and radiofrequency (6.4%). Injectable modalities (IRAP, PRP, mesotherapy, stem cells) were almost solely administered by veterinarians; other modalities were variably applied by veterinarians, technicians, veterinary assistants, farriers, physical therapists, trainers, and other entities. A total of 33% of respondents reported working collaboratively with physical therapists on equine patients. Findings indicate that a broad range of invasive and non-invasive modalities are used in equine patients to address a variety of rehabilitation and performance needs, and that personnel with varying levels of expertise are involved in their administration. This suggests that further investigation to better define the delivery, efficacy and any negative effects of many of these modalities is important.

## Introduction

In the last two decades, the practice of equine sports medicine and rehabilitation has progressively developed into a large and focused field of specialized equine practice (1–3). Reflecting this phenomenon is the growth of organized training programs, and the consolidation of expert practitioners into groups with objectives that include sharing of and furthering knowledge in this field. Relevant examples include the Equine Rehabilitation Certificate Program (CERP), established in 2004, which offers training in equine rehabilitation for veterinarians, veterinary technicians, physical therapists, and assistants, and students of these fields. The American College of Veterinary Sports Medicine and Rehabilitation (ACVSMR) was approved by the American Veterinary Medical Association in 2010 and currently comprises over 100 equine interest diplomates and veterinarians in residency training programs distributed across multiple countries. In addition, other national and international groups contain practitioners who utilize rehabilitation modalities in equine practice, including the International Society of Equine Locomotor Pathology (ISELP, established 2007) with over 600 members, and the American Association of Rehabilitation Veterinarians (AARV). Physical therapists have also displayed a burgeoning interest in animal rehabilitation, and both the Orthopedic Section of the American Physical Therapy Association and the World Confederation for Physical Therapy have established subgroups for physical therapists with interest and expertise in animal rehabilitation (4). Furthermore, in 2016 the American Veterinary Medical Association published a resolution for the incorporation of Complementary Alternative Veterinary Medicine into the veterinary curriculum, which encompasses commonly used rehabilitation modalities such as acupuncture and chiropractic treatment (5).

Accompanying this growing field of practice, a progressively numerous array of rehabilitation modalities and techniques are available for use by veterinarians, physiotherapists, and lay persons (1, 3, 6, 7). A frequent objective of the use of these modalities is to facilitate return to performance after injury. To be achieved successfully, this requires accurate diagnosis of the injured tissue, selection of appropriate modalities in treatment, and regular assessment of the healing response during therapy. Different modalities are utilized to variably assist with pain reduction, restoration of range of motion, and healing and strengthening of tissues to restore health and performance.

Rehabilitation modalities and techniques can be broadly divided into categories, including specific exercise activities, thermal modalities, electrophysical modalities, biologic modalities, and acupuncture, and mechanical soft tissue modalities. Other available modalities include mesotherapy, hyperbaric oxygen therapy, and therapeutic shoeing.

Controlled or targeted exercise is a common foundation for most equine rehabilitation programs (8). Controlled walking activity (in hand or automatic horse walker) is used to improve mobility and reduce swelling, assist in tissue repair by facilitating tendon fiber alignment, preventing restrictive adhesions and promoting a gradual return of cardiovascular fitness and bone strength (1). As healing progresses, more intensive exercise activities can be applied, which can include specific therapeutic limb and core exercises with or without the incorporation of resistance bands, taping, or limb stimulators; swimming, and treadmill exercise (aqua and land) to optimize strength, flexibility, and fitness.

Thermal therapy can include cooling modalities (cold-water circulation machine, cold-water hydrotherapy, ice) to reduce pain, decrease tissue swelling, and alter circulation (1). Heat can be applied to increase local circulation, muscle relaxation, and tissue extensibility (1).

Electrophysical modalities include shockwave (focused and radial), Transcutaneous Electrical Nerve Stimulation (TENS), Neuromuscular Electrical Stimulation (NMES), Pulsed Electromagnetic Field Therapy (PEFM), therapeutic ultrasound, cytowave, radiofrequency therapy, cold laser, and vibration therapy (7, 9). These modalities variably provide analgesia and reduce tissue edema, fibrous scar formation, and inflammatory mediators, and may counteract disuse atrophy (1, 6, 9). Shockwave therapy is proposed to increase tissue concentrations of angiogenic cytokines, growth factors, osteoblasts, and mesenchymal stem cells (1, 9).

Biologic modalities (PRP, IRAP/autologous conditioned serum, and stem cells) aim to repair damaged tissue, inhibit inflammatory cascades and encourage tissue regeneration (10). Mesotherapy also represents an injection-based technique that aids in localized pain reduction (11).

A variety of mechanical techniques can impact soft tissues and joints, including compression bandaging, massage, stretching, therapeutic exercises, taping, and chiropractic or joint mobilization (12, 13). These can be used to reduce pain and inflammation, enhance tissue repair, improve soft tissue extensibility and function, reduce muscle hypertonicity, and increase range of motion. Acupuncture aids in pain control, reduces edema, muscle spasm and scar tissue, and promotes vasodilation and neuronal regeneration (14).

Finally, therapeutic shoeing can help displace concussion, change the traction between the ground and the shoe, alter the flight phase of the stride, distribute force, move the center of pressure of the hoof, and change the movement of the distal interphalangeal joint (15).

Since clearly a wide array of rehabilitation modalities are available to current day practitioners, the objective of this study was to determine which modalities are commonly used in equine practice by surveying a number of national and international equine veterinary groups containing members practicing equine sports medicine and rehabilitation. Furthermore, it was intended to determine how modalities are applied in the management of performance, disease, or injury in athletic horses, and which operators are providing the modalities. Such information is valuable in determining which modalities would benefit most from rigorous assessments of their efficacy and risks, ultimately to improve the burgeoning practice of equine rehabilitation.

## Materials and methods

An electronic questionnaire querying the use of 38 different rehabilitation modalities was distributed to veterinarians in eight national and international veterinary groups and associations^1^. The questionnaire link was distributed between August 2016 through January 2017 via email lists for diplomates of the American College of Veterinary Internal Medicine (ACVIM) and the ACVSMR. The announcement and link were also emailed to the equine client list of a shockwave company^2^, and to the members of the International Show Horse Veterinary Association (ISHVA). The questionnaire link was published in the newsletters of the International Society of Equine Locomotor Pathology (ISELP) and the American Association of Equine Practitioners (AEEP). Additionally, the questionnaire link was posted on the websites of the British Equine Veterinary Association (BEVA) and the Australian Equine Veterinary Association (AEVA). Contacting the membership of the American College of Veterinary Surgeons (ACVS) *en masse* was unable to be accomplished due to their diplomate contact policies. Although an exact number of individuals receiving the questionnaire could not be documented, based on approximate sizes of the groups that were contacted, it is estimated that the questionnaire was distributed to more than 2,000 equine veterinarians. Each respondent was given a unique identifier to avoid duplication of answers. The project did not meet the definition of “human subject” under the Common Rule (45 CFR 46) and therefore did not require review by the ethics committee at Oregon State University that oversees research on humans.

Respondents were queried regarding their form of practice and their geographic location; the equine athletic disciplines and breeds they most commonly work with; and the specific rehabilitation modalities they employ from a list of 38 proffered modalities contained within the survey (Table 1). Additionally, respondents were queried regarding a list of eight broad conditions or situations that are commonly encountered in horses in which rehabilitation modalities might be considered important. These included tendon or ligament injury, injury to the neck or back, generalized muscle strain, application after fracture repair, arthroscopy, or colic surgery, and for addressing poor performance or for maintaining performance. Respondents were also queried regarding the personnel involved in administering the modalities in question, selecting from the options of licensed veterinarian, licensed veterinary technician, veterinary assistant, veterinary student, farrier, licensed physical therapist, layperson, and trainer/owner. Responses were collated and evaluated by topic (question). No statistical measures were undertaken.

**Table 1:** Rehabilitation modalities listed in descending order of utilization by responding practitioners.

**Modality**	**Proportion of respondents that use each modality (%)**	**Total number of responses for each modality**
Controlled hand walking	97.3	258
Therapeutic shoeing	96.1	257
Ice	95.2	248
Compression bandaging	89.5	247
Platelet rich plasma	86.5	251
Therapeutic exercises	84.3	242
Stretching	83.3	239
Cold water hydrotherapy	82.9	228
IRAP	81.4	247
Heat	77.6	232
Chiropractic	72.8	243
Shockwave therapy - focused	72.4	243
Range of motion therapy	71.9	235
Massage	69.0	232
Acupuncture	68.3	243
Stem cells—mesenchymal	62.7	233
Automatic horse walker	56.7	238
Mesotherapy	56.4	236
Cold water circulation machine	48.5	231
Pessoa® lunging system	46.2	225
Treadmill—land	39.9	218
Vibration therapy	39.6	220
Class 4 cold laser	39.2	227
Therapeutic ultrasound	39.0	223
Treadmill—aqua	39.0	223
Stem cells—adipose	36.6	216
Class 3 or less cold laser	34.3	219
Kinesio taping®	33.0	218
Neuromuscular electrical stimulation (NMES)	31.8	217
Swimming	30.4	217
Transcutaneous electrical nerve stimulation (TENS)	29.2	216
Shockwave therapy—radial	28.6	217
Equiband™	27.4	215
Pulsed electromagnetic field therapy (PEMF)	22.9	214
Saltwater spa	21.1	213
Hyperbaric oxygen chamber	9.4	203
Cytowave	8.3	206
Radiofrequency therapy	6.4	203

## Results

A total of 305 complete (*n* = 175) or partial (*n* = 130) survey responses were obtained from over 10 geographic regions. Partial responses represented those in which not every question in the survey was answered. A total of 75.4% of surveys were submitted from private equine practice or regional private equine referral hospitals, 14.1% from university teaching hospitals or satellite clinics, 8.2% from private mixed animal practice, and 2.3% from veterinary rehabilitation centers. The majority of respondents were located in the USA (60%), Europe (25.6%), and Canada (5.6%), followed by Australia/South Pacific (3.6%), Middle East (2.0%), and Central America (1%); the remaining few respondents were located in South America, Africa, Asia, and the Caribbean.

**Figure 1:**
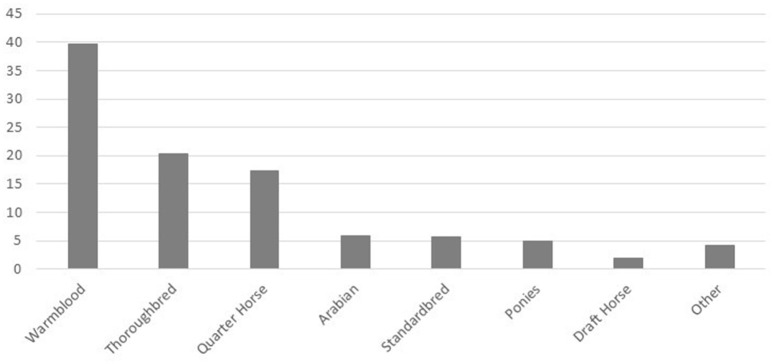
Proportion (%) of horses of various breeds presenting to responding practitioners.

**Figure 2:**
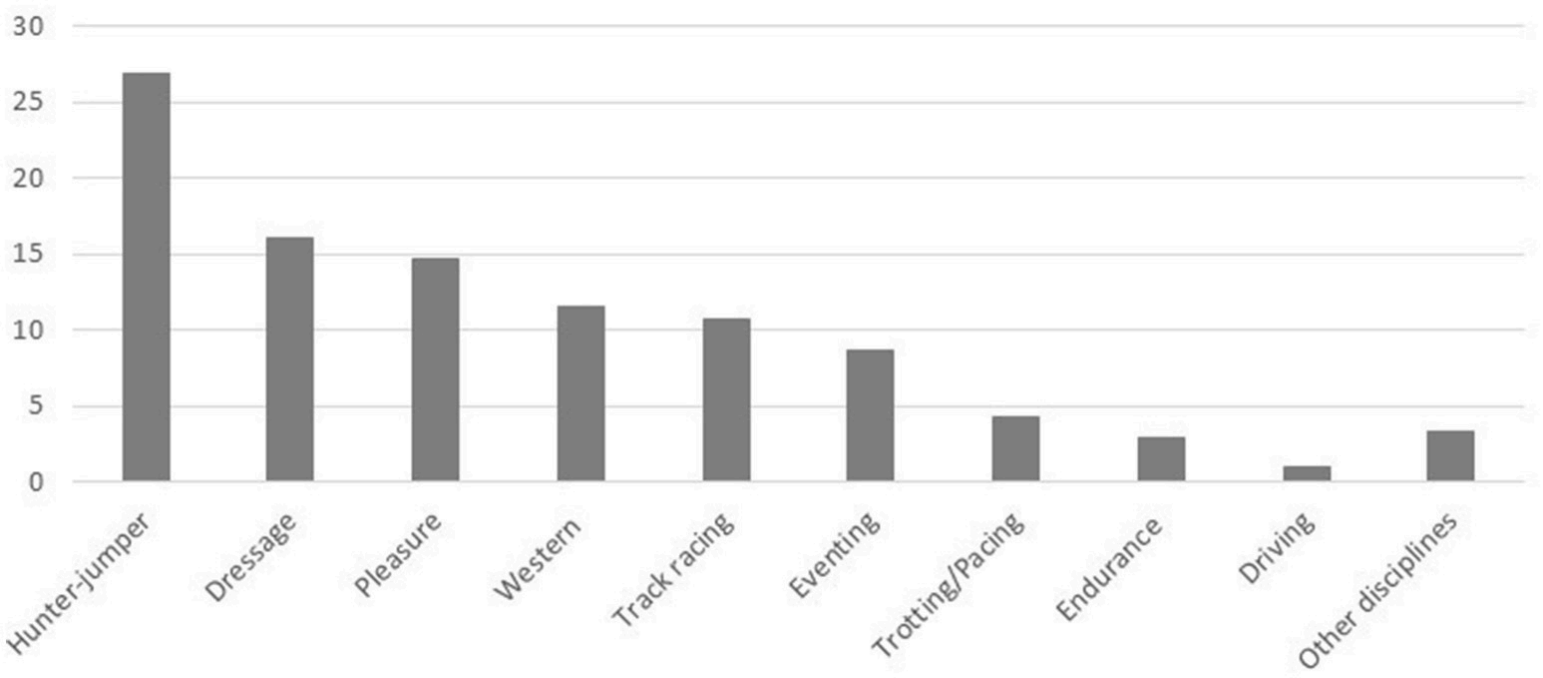
Proportion (%) of horses in various athletic disciplines presenting to responding practitioners.

Respondents reported by percentage, the proportion of horses of specific breeds that presented to them annually (Figure 1), and the specific athletic disciplines the horses they worked with participated in (Figure 2). In regard to the proportion of specific equine breeds that respondents (*n* = 267) interacted with, Warmbloods figured most prominently, followed by Thoroughbreds and Quarter horses, with lesser proportions of Arabians, Standardbreds, ponies, Draft horses, and other breeds (Figure 1). Respondents (*n* = 243) were most commonly treating horses associated with the disciplines of hunter-jumper, dressage, pleasure riding, Western riding, track racing, and eventing (Figure 2). A smaller proportion of respondents also worked with horses in trotting or pacing, endurance, driving, and other disciplines (Figure 2).

Of the 38 rehabilitation modalities that were presented to respondents, the most prominently utilized (those that were used by the highest proportion of respondents regardless of conditions treated) included controlled hand walking, therapeutic shoeing, and ice, which were used by more than 90% of respondents (Table 1). Compression bandaging, PRP, specific therapeutic exercises, interleukin-1 receptor antagonist protein therapy (IRAP), stretching, and cold water hydrotherapy were used by more than 80% of respondents (Table 1). The remaining modalities were utilized by between 6 and 78% of respondents, with hyperbaric oxygen chamber (9.4%), cytowave (8.3%), and radiofrequency (6.4%) representing the least selected modalities (Table 1).

In regard to acupuncture and soft tissue modalities (including chiropractic, stretching, range of motion, and massage; Table 2), chiropractic was most often applied to horses for maintenance of performance or addressing poor performance, followed by injuries of the neck or back and generalized muscle strain. Acupuncture, massage, and stretching were similarly most commonly applied to these same four situations, though stretching was more commonly applied for tendon and ligament injuries than for poor performance (Table 2). Range of motion therapy was equivalently applied most often for tendon and ligament injuries and injuries of the neck or back, and also for generalized muscle strain. Compression bandaging was typically applied to tendon and ligament injuries and after arthroscopy (Table 2).

**Table 2:** Utilization of acupuncture and mechanical soft tissue modalities by practitioners for 8 different medical scenarios.

**Acupuncture & soft tissue modalities**	**Compression bandaging (%)**	**Stretching (%)**	**Chiropractic (%)**	**Acupuncture (%)**	**Range of motion (%)**	**Massage (%)**
Tendon or ligament injury	91.4	54.8	17.8	27.2	66.2	33.6
Neck or back injury	4.8	74.4	72.0	74.2	66.9	71.8
Generalized muscle strain	19.3	81.6	66.9	83.7	63.3	90.1
Post fracture repair	41.2	20.8	5.7	10.2	39.6	12.2
Post arthroscopy	50.3	25.0	8.9	11.6	46.0	12.2
Post colic surgery	11.8	14.9	10.2	38.1	10.1	9.9
Poor performance	8.0	49.4	77.7	67.4	36.7	55.0
Maintain performance	25.7	66.7	81.5	75.5	52.5	69.5
Other (not specified)	16.6	16.1	17.8	23.8	15.8	14.5
Total responses per modality	187	168	157	147	139	131

In regard to electrophysical modalities, laser, therapeutic ultrasound, and focused shockwave were predominantly applied to tendon and ligament injuries, though injuries of the neck and back, and generalized muscle strain also represented commonly treated conditions (Table 3). Radial shockwave was most often applied to neck and back injuries and tendon or ligament injuries, while NMES, TENS, and PEMF were applied most often to neck and back injuries and for generalized muscle strain (Table 3).

**Table 3:** Utilization of electrophysical modalities by practitioners for 8 different medical scenarios.

**Electrophysical modalities**	**Shockwave (focused) (%)**	**Class 4 laser (%)**	**Therapeutic ultrasound (%)**	**≤ Class 3 laser (%)**	**Shockwave (radial) (%)**	**NMES (%)**	**TENS (%)**	**PEMF (%)**
Tendon or ligament injury	95.4	96.3	93.2	86.4	66.7	25.0	42.9	59.5
Neck or back injury	71.7	60.5	54.1	53.0	72.2	69.2	77.6	67.6
Generalized muscle strain	35.5	65.4	51.4	62.1	38.9	59.6	63.3	62.2
Post fracture repair	10.5	25.9	9.5	13.6	1.9	7.7	10.2	35.1
Post arthroscopy	6.6	27.2	6.8	16.7	3.7	3.9	8.2	35.1
Post colic surgery	0.0	18.5	5.4	6.1	0.0	5.8	10.2	10.8
Poor performance	15.8	27.2	12.2	25.8	20.4	28.9	18.4	27.0
Maintain performance	30.3	28.4	24.3	27.3	29.6	30.8	34.7	43.2
Other (not specified)	19.7	16.1	12.2	16.7	9.3	23.1	30.6	18.9
Total responses per modality	152	81	74	66	54	52	49	37

*NB cytowave and radiofrequency omitted due to minimal number of responses*.

Biologic modalities, including stem cells (adipose and mesenchymal), PRP, and IRAP were overwhelmingly directed at the treatment of tendon and ligament injury, though they were also frequently used following arthroscopic surgery, with IRAP divided equivalently between these situations (Table 4). Mesotherapy was largely applied to injuries of the back and neck, and to generalized muscle strain (Table 4).

**Table 4:** Utilization of biologic and injectable modalities by practitioners for 8 different medical scenarios.

**Biologic and injectable modalities**	**PRP (%)**	**IRAP (%)**	**Stem cells (mesenchymal) (%)**	**Mesotherapy(%)**	**Stem cells (adipose) (%)**
Tendon or ligament injury	98.9	54.0	98.3	1.8	95.1
Neck or back injury	13.0	18.6	11.9	87.4	16.4
Generalized muscle strain	1.6	3.1	1.7	57.7	1.6
Post fracture repair	6.0	6.8	10.2	1.8	6.6
Post arthroscopy	24.3	55.3	41.5	0.9	34.4
Post colic surgery	0.0	0.0	0.0	1.8	0.0
Poor performance	5.4	8.7	2.5	31.5	3.3
Maintain performance	12.4	32.3	4.2	33.3	4.9
Other (not specified)	10.8	30.4	14.4	10.8	21.3
Total responses per modality	185	161	118	111	61

Therapeutic shoeing was used by a large number of respondents (*n* = 210) primarily for the purpose of addressing tendon and ligament injury (92.4% of responses) but also for maintenance of performance (50.5% of responses) ahead of other specified uses (range 0–25%). Vibration therapy was also used for these two conditions (65.8 and 57.9% of 76 responses, respectively) as well as for generalized muscle strain (54.0%) ahead of other specified uses (range 13.2–47.4%). Kinesiotaping® was utilized equivalently (72.4% of 58 responses) for both neck and back injury and generalized muscle strain, and less often for all other specified conditions (range 13.8–51.7%). Few respondents (*n* = 16) utilized hyperbaric oxygen chambers, however, of those, half applied it for tendon and ligament injury, and one quarter used it after colic surgery.

Cold thermal modalities (cold water hydrotherapy, cold water circulation machine, saltwater spa, and ice) were commonly applied to tendon and ligament injury, generalized muscle strain and to maintain performance, whereas heat was most often applied to generalized muscle strain, followed by neck or back injury and tendon and ligament injury.

Exercise based modalities (walking, therapeutic exercises, Pessoa® lunging system, treadmill, swimming, and Equiband™) were variably applied in all medical scenarios, particularly in tendon and ligament injury, and neck or back injury.

Regarding the administration of modalities, respondents were asked to indicate which personnel provided each of the 38 different modalities. Responding veterinarians indicated that they were the personnel providing injection based modalities in most cases, including stem cells (mesenchymal: 99.1% of *n* = 112 selections in this category; adipose: 100%, *n* = 59), PRP (98.3%, *n* = 175), IRAP (97.5%, *n* = 161), and mesotherapy (95.4%, *n* = 105). Veterinarians were also the most frequent administrators of acupuncture (88.5%, *n* = 148) and chiropractic (69.8%, *n* = 162). Veterinarians were the most common personnel to apply shockwave therapy (focused: 65.4%, *n* = 202; radial: 62.5%, *n* = 59), and therapeutic ultrasound (31.9%, *n* = 138), followed by licensed veterinary technicians (17.3, 16.7, and 20.3% for the three modalities, respectively). Veterinarians were also the main providers of class 4 laser (47.2%, *n* = 123), followed by veterinary technicians (25.2%) or veterinary assistants (18.7%), with limited provision of this modality by other types of personnel. Class 3 or less laser was provided by veterinary technicians (17.1%, *n* = 105 total responses), owners or trainers (15.2%), and veterinary assistants (14.3%) behind veterinarians (36.2%). Veterinarians less often, but as the most frequent personnel, administered NMES (54.3%, *n* = 70), kinesiotaping® (52.7%, *n* = 74), TENS (43.2%, *n* = 88), and hyperbaric oxygen (40.9%, *n* = 22), as well as compression bandaging (31.5%, *n* = 378) and PEMF (23.8%, *n* = 63).

Farriers were the personnel most likely to provide therapeutic shoeing (82.7%, *n* = 226) followed by veterinarians (14.2%), and farriers rarely administered any other modality. Licensed physical therapists were common providers of massage (26.1%, *n* = 180), chiropractic (17.9%, *n* = 162), kinesiotaping® (16.2%, *n* = 74), NMES (14.3%, *n* = 70), and TENS (12.5%, *n* = 88). Veterinary students were common providers of thermal modalities and walking.

Lay people were common providers of walking (in-hand or automatic walker), massage, swimming, treadmill (land and aqua), vibration therapy, cold water modalities, and PEMF. Lay people also reportedly provided some chiropractic therapy (8.0%). Trainers and owners similarly administered most of these modalities, and more commonly than lay people were also reported to administer or use the Pessoa® system (55.5%, of *n* = 128 total selections), Equiband™ (37.0%, *n* = 73), heat (33.7%, *n* = 294), ice (31.5%, *n* = 429), stretching (31.1%, *n* = 360), cold water hydrotherapy (30.3%, *n* = 330), compression bandaging (22%, *n* = 378), and therapeutic ultrasound (16.0%, *n* = 138).

## Discussion

A large array of non-invasive and invasive modalities are utilized in the treatment or management of a wide range of disorders affecting equine athletes in many disciplines. The findings from this survey confirm that a range of rehabilitation modalities are commonly implemented for a variety of conditions in athletic horses, and, according to responding veterinarians in this survey, many of these modalities are administered by personnel with a wide range of experience and expertise.

The most commonly utilized modalities, selected by more than three-quarters of respondents, are considered relatively innocuous, and included controlled hand walking, thermal modalities (ice, cold water hydrotherapy, and heat), compression bandaging, therapeutic exercises, and stretching. These modalities have the advantages of being relatively inexpensive, and can be applied by personnel with relatively basic levels of expertise or experience. Nonetheless, they can be time consuming, and present some risk if inappropriately applied or inadequately supervised. Veterinarians have a crucial role in educating personnel involved in these activities, and in helping establish organized and progressive rehabilitation programs which incorporate appropriate modalities for the condition being addressed.

Therapeutic shoeing was the second most commonly selected modality in the survey, and veterinarians indicated that this modality was extremely likely to be provided by farriers. Given the importance of therapeutic shoeing as a modality, particularly in horses with laminitis or other challenging foot, ligament or tendon conditions, collaborative efforts between veterinarians, and farriers are critical. Attention to fostering this relationship must be emphasized in the curricula for both professions (16, 17).

The administration of PRP and IRAP were also among the most commonly used modalities, with over 80% of responding veterinarians indicating their use. In a previous very large survey of medical joint modalities used by practitioners in 2009, ~54% of veterinarians indicated that they used IRAP products (18). It is possible that the higher affirmative response in the current survey might reflect increasing use of this modality, a higher percentage of specialist practitioners in the current survey population, recent development of a potentially more efficacious version (IRAP II), or the equine disciplines or expectations of the clients the respondents work with (18, 19). In the previously mentioned study, English performance horse veterinarians were reported to be more likely to use IRAP than either show horse or racehorse veterinarians, though it was not clear if this related to different pathologies encountered in the different disciplines (18). In the current study, IRAP was directed equivalently toward tendon and ligament injury, and after arthroscopy, both of which are indicated uses at this time (20, 21).

Tendon injuries in particular may also be treated with PRP, however, the utilization of PRP is complicated by critical issues such as methods of preparation and platelet activation, with a large array of commercial systems to select from (22, 23). Since PRP is one of the most commonly utilized modalities by sports medicine practitioners it is critical that they have a thorough understanding of the advantages and limitations of the available systems to optimize the likelihood of a positive outcome in their patients.

Manual modalities (chiropractic, massage, range of motion) and acupuncture were commonly utilized modalities in this survey. Chiropractic, massage and acupuncture all commonly fall under the practice of licensed veterinarians, though this requirement can vary between states and countries (24). Reflecting this phenomenon, acupuncture was reportedly typically provided by licensed veterinarians in this survey, and the majority of chiropractic was administered by veterinarians or physical therapists. Physical therapists were reported to be the most common providers of massage. A total of 33% of veterinarians responding to the survey indicated that they consult with physical therapists certified in veterinary rehabilitation, supporting the concept that this is a growing and important collaborative relationship within the practice of equine sports medicine (4). Responding veterinarians indicated that lay people also provided some chiropractic (8%) as well as massage (19.4%). Practice of these techniques by laypeople is becoming more frequent, and has been a source of conflict, because it can potentially create risk to patients through iatrogenic injury, particularly if administration of sedative drugs by unlicensed personnel occurs (24). Furthermore, chiropractic and acupuncture were both commonly utilized for the treatment of poor performance in this survey, and when not performed by a veterinarian, the opportunity to identify specific disease conditions creating performance issues is substantially reduced. Veterinarians should ensure that owners and trainers understand the legal boundaries for specific activities, and should consider establishing collaborative supervisory relationships to facilitate delivery of modalities that they themselves may not provide. Incorporation of appropriate training in alternative modalities into the professional curriculum as recently proposed also enhances the probability of new graduates entering practice with a solid understanding of how to manage specific modalities (5, 25). Furthermore, ensuring that veterinary students and veterinarians in training programs receive adequate practical training in relevant modalities is critical. The results of the current survey suggest that veterinary students were most likely to be involved in delivery of very basic modalities including walking and thermal modalities. Increased emphasis on including rehabilitative and alternative modalities in the professional curriculum will hopefully also translate to more practical training opportunities in more advanced modalities prior to graduation.

The selection of modalities by practitioners in this survey was likely strongly influenced by a range of factors, including cost, convenience, access, and personal capabilities and bias. Some modalities require expensive equipment or are labor intensive, requiring additional personnel to support their administration. Others require substantial training and experience, such as acupuncture and chiropractic. Access and regulation is another influencing variable, which has most recently impacted stem cell therapy in the USA. The Food and Drug Administration (FDA) has classified stem cells as a “new animal drug” therefore requiring FDA approval prior to use for treatment purposes or in live animal research^3^ Furthermore, certain modalities, such as shockwave, may be banned from some competitive events for a specified period of time ahead of competition. Ideally, the selection of modalities should primarily be driven by known or proven efficacy, which in itself is a dynamic characteristic since techniques and equipment evolve, improve, or are disproved (7, 26–28). The personal preferences of respondents for specific modalities represented in this survey may also be a source of bias in the data since one of the contact lists utilized was the client list of a shockwave company.

In conclusion, the survey findings indicate that a wide range of rehabilitation modalities are commonly utilized in athletic horses for a variety of reasons, and are administered by a variety of personnel. Additional investigations better defining the utility and efficacy of most of these modalities is indicated, particularly those that are utilized the most frequently or which are associated with considerable expense or the risk of adverse effects on equine patients.

## Ethics statement

Data was collected via means of electronic survey where respondents were provided complete anonymity and could not be identified by the investigators or others. The project did not meet the definition of “human subject” under the Common Rule (45 CFR 46) and therefore did not require review by the ethics committee at Oregon State University that oversees research on humans.

## Author contributions

JW and EM were responsible for study design and data collection. JW, EM, and KD-Z contributed to data assessment and manuscript preparation.

### Conflict of interest statement

The authors declare that the research was conducted in the absence of any commercial or financial relationships that could be construed as a potential conflict of interest.

## Footnotes

^1^Survey monkey—Surveymonkey.com, Portland, Oregon.

^2^PulseVet—https://www.pulsevet.com/

^3^Guidance for Industry. Cell-Based Products for Animal Use. June 2015 https://www.fda.gov/downloads/AnimalVeterinary/GuidanceComplianceEnforcement/GuidanceforIndustry/UCM405679.pdf.

## References

[1] Kaneps AJ. Practical rehabilitation and physical therapy for the general equine practitioner. Vet Clin North Am Equine Pract. (2016) 32:167–80. 10.1016/j.cveq.2015.12.001 26898959

[2] King MR Davidson EJ. Innovations in equine physical therapy and rehabilitation. Vet Clin North Am Equine Pract. (2016) 32:xiii–xiv. 10.1016/j.cveq.2016.02.001 27012510

[3] Bergh A Physical treatment of the equine athlete. In: Hinchcliff KW Kaneps AJ Geor RJ editors. Equine Sports Medicine and Surgery, 2nd Edn. China: Saunders Elsevier (2014). p. 1231–41.

[4] McGowan CM Cottriall S. Introduction to equine physical therapy and rehabilitation. Vet Clin North Am Equine Pract. (2016) 32:1–12. 10.1016/j.cveq.2015.12.006 26906262

[5] Memon MA Shmalberg J Adair HS Allweiler S Bryan JN Cantwell S. Integrative veterinary medical education and consensus guidelines for an integrative veterinary medicine curriculum within veterinary colleges. Open Vet J. (2016) 6:44–56. 10.4314/ovj.v6i1.7 27200270 PMC4824037

[6] Porter M. Equine rehabilitation therapy for joint disease. Vet Clin North Am Equine Pract. (2005) 21:599–607. 10.1016/j.cveq.2005.08.002 16297723

[7] Buchner HH Schildboeck U. Physiotherapy applied to the horse: a review. Equine Vet J. (2006) 38:574–80. 10.2746/042516406X153247 17124850

[8] Gillis C Soft tissue injuries: tendinitis and desmitis. In: Hinchcliff KW Kaneps AJ Geor RJ editors. Equine Sports Medicine and Surgery, 2nd Edn. China: Saunders Elsevier (2014). p. 399–418.

[9] Schlachter C Lewis C Electrophysical therapies for the equine athlete. veterinary clinics of north america: equine practice. rehabilitation of the equine athlete. Elsevier (2016) 32:127–47. 10.1016/j.cveq.2015.12.011 27012509

[10] Broeckx S Zimmerman M Crocetti S Suls M Mariën T Ferguson SJ. Regenerative therapies for equine degenerative joint disease: a preliminary study. PLoS ONE (2014) 9:e85917. 10.1371/journal.pone.0085917 24465787 PMC3896436

[11] Massimo M Gatti A Maggiori S Alessandro S Role of mesotherapy in musculoskeletal pain. opinions from the Italian society of mesotherapy. Evid Based Complement Alternat Med. (2012) 2012:436959 10.1155/2012/436959 22654954 PMC3359685

[12] Haussler KK Review of manual therapy techniques in equine practice. J Equine Vet Sci. (2009) 29:849–69. 10.1016/j.jevs.2009.10.018

[13] Haussler K. Joint Mobilization and manipulation for the equine athlete. Vet Clin North Am Equine Pract. (2016) 32:87–101. 10.1016/j.cveq.2015.12.003 27012508

[14] le Jeune S Henneman K May K. Acupuncture and equine rehabilitation. Vet Clin North Am Equine Pract. (2016) 32:73–85. 10.1016/j.cveq.2015.12.004 26906261

[15] Parks A. Therapeutic farriery. one veterinarian's perspective. Vet Clin North Am Equine Pract. (2012) 28:333–50. 10.1016/j.cveq.2012.05.003 22981193

[16] Baker WRJr. Treating laminitis: beyond the mechanics of trimming and shoeing. Vet Clin North Am Equine Pract. (2012) 28:441–55. 10.1016/j.cveq.2012.05.004 22981200

[17] Moyer W O'Grady SE Werner HW. The equine practitioner-farrier relationship: building a partnership. Vet Clin North Am Equine Pract. (2012) 28:117–29. 10.1016/j.cveq.2012.03.003 22640583

[18] Ferris DJ Frisbie DD McIlwraith CW Kawcak CE. Current joint therapy usage in equine practice: a survey of veterinarians 2009. Equine Vet J. (2011) 43:530–5. 10.1111/j.2042-3306.2010.00324.x 21668486

[19] Hraha TH Doremus KM McIlwraith CW Frisbie DD. Autologous conditioned serum: the comparative cytokine profiles of two commercial methods (IRAP and IRAP II) using equine blood. Equine Vet J. (2011) 43:516–21. 10.1111/j.2042-3306.2010.00321.x 21496084

[20] Geburek F Lietzau M Beineke A Rohn K Stadler PM. Effect of a single injection of autologous conditioned serum (ACS) on tendon healing in equine naturally occurring tendinopathies. (2015) Stem Cell Res Ther. 6:126. 10.1186/s13287-015-0115-0 26113022 PMC4513386

[21] McIlwraith CW Wright I Nixon AJ. (eds). Post-operative management, adjunctive therapies and rehabilitation procedures. In: Diagnostic and Surgical Arthroscopy in the Horse, 4th Edition. China: Mosby Ltd (2014). p. 444.

[22] Textor J. Autologous biologic treatment for equine musculoskeletal injuries: platelet-rich plasma and IL-1 receptor antagonist protein. Vet Clin North Am Equine Pract. (2011) 27:275–98. 10.1016/j.cveq.2011.05.001 21872759

[23] Romero A Barrachina L Ranera B Remacha AR Moreno B de Blas I. Comparison of autologous bone marrow and adipose tissue derived mesenchymal stem cells, and platelet rich plasma, for treating surgically induced lesions of the equine superficial digital flexor tendon. Vet J. (2017) 224:76–84. 10.1016/j.tvjl.2017.04.005 28697880

[24] Macejko C State Boards Wage War on Lay Persons Practicing Veterinary Medicine. 01:2008 *By DVM360 MAGAZINE* (2008). Available online at: http://veterinarynews.dvm360.com/state-boards-wage-war-lay-persons-practicing-veterinary-medicine

[25] Memon MA Sprunger LK. Survey of colleges and schools of veterinary medicine regarding education in complementary and alternative veterinary medicine. J Am Vet Med Assoc. (2011) 239:619–23. 10.2460/javma.239.5.619 21879961

[26] Koch TG Berg LC Betts DH. Concepts for the clinical use of stem cells in equine medicine. Can Vet J. (2008) 49:1009–17. 10.1016/j.rvsc.2018.03.011 19119371 PMC2553494

[27] Montgomery L Elliott SB Adair HS. Muscle and tendon heating rates with therapeutic ultrasound in horses. Vet Surg. (2013) 42:243–9. 10.1111/j.1532-950X.2013.01099.x 23373839

[28] Duesterdieck-Zellmer KF Larson MK Plant TK Sundholm-Tepper A Payton ME. (2016). *Ex vivo* penetration of low-level laser light through equine skin and flexor tendons. Am J Vet Res. 77:991–9. 10.2460/ajvr.77.9.991 27580111

